# LoViT: Long Video Transformer for surgical phase recognition

**DOI:** 10.1016/j.media.2024.103366

**Published:** 2025-01

**Authors:** Yang Liu, Maxence Boels, Luis C. Garcia-Peraza-Herrera, Tom Vercauteren, Prokar Dasgupta, Alejandro Granados, Sébastien Ourselin

**Affiliations:** a Department of Surgical & Interventional Engineering, King’s College London, United Kingdom; b Department of Peter Gorer Department of Immunobiology, King’s College London, United Kingdom

**Keywords:** Surgical phase recognition, Long videos, Temporally-rich spatial feature, Multi-scale, Phase transition-aware

## Abstract

Online surgical phase recognition plays a significant role towards building contextual tools that could quantify performance and oversee the execution of surgical workflows. Current approaches are limited since they train spatial feature extractors using frame-level supervision that could lead to incorrect predictions due to similar frames appearing at different phases, and poorly fuse local and global features due to computational constraints which can affect the analysis of long videos commonly encountered in surgical interventions. In this paper, we present a two-stage method, called Long Video Transformer (LoViT), emphasizing the development of a temporally-rich spatial feature extractor and a phase transition map. The temporally-rich spatial feature extractor is designed to capture critical temporal information within the surgical video frames. The phase transition map provides essential insights into the dynamic transitions between different surgical phases. LoViT combines these innovations with a multiscale temporal aggregator consisting of two cascaded L-Trans modules based on self-attention, followed by a G-Informer module based on *ProbSparse* self-attention for processing global temporal information. The multi-scale temporal head then leverages the temporally-rich spatial features and phase transition map to classify surgical phases using phase transition-aware supervision. Our approach outperforms state-of-the-art methods on the Cholec80 and AutoLaparo datasets consistently. Compared to Trans-SVNet, LoViT achieves a 2.4 pp (percentage point) improvement in video-level accuracy on Cholec80 and a 3.1 pp improvement on AutoLaparo. Our results demonstrate the effectiveness of our approach in achieving state-of-the-art performance of surgical phase recognition on two datasets of different surgical procedures and temporal sequencing characteristics. The project page is available at https://github.com/MRUIL/LoViT.

## Highlights

• A temporally-rich spatial feature extractor to extract spatial feature.
• Multi-scale temporal feature aggregation of local and global relationships.
• A phase transition-aware supervision for highlighting phase transitions.

## 1 Introduction

The growing field of surgical data science (SDS) aims to transform interventional healthcare through the advanced use of data collected from medical devices within the operating room (OR) (Maier-Hein et al., 2022). Central to SDS is the classification and understanding of surgical workflows, critical not only for the understanding of surgical procedures, but also for the evaluation of surgical skills and the provision of context-sensitive intraoperative support (Vercauteren et al., 2020). The automation of surgical phase and action identification is paramount in this regard, enhancing surgical proficiency, safety, and overall operational efficacy by enabling the provision of real-time feedback to the surgical team. Such advancements lay the groundwork for the continuous evolution of surgical techniques and training methodologies.

In the realm of endoscopic surgery, the task of surgical phase recognition entails segmenting video frames into distinct operational stages, providing a high-level overview of the procedure (Garrow et al., 2021). This classification focuses on identifying the broader phases of surgery. Conversely, action recognition within this context delves into a more detailed analysis, pinpointing specific tasks and actions within individual frames.

Early work in automated surgical phase recognition (Blum et al., 2010, Bardram et al., 2011, Dergachyova et al., 2016, Jin et al., 2018, Gao et al., 2021, Quellec et al., 2015, Holden et al., 2014) primarily utilized statistical models and additional data, such as annotations or tool-related information, showcasing proficiency within the intricate realm of surgical video analytics, although they exhibit limited representational capacities due to predetermined dependencies (Jin et al., 2018, Gao et al., 2021). With the advent of deep learning, a shift towards exclusively video-centric approaches has become evident. Multifaceted learning strategies that require annotations for both tools and phases have been explored, thus increasing the annotation burden (Twinanda et al., 2017, Twinanda, 2017, Jin et al., 2020). Yet, the current trajectory leans towards single-task learning, which we further investigate in our study.

**Fig. 1:**
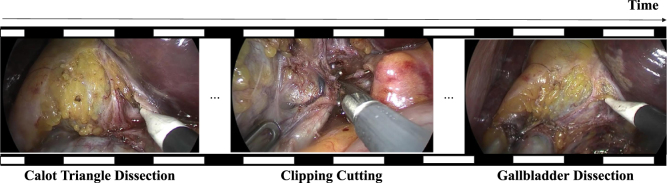
Example of similar frames (first and third) corresponding to different phases in Cholec80 dataset (Twinanda et al., 2017).

Constructing an end-to-end model capable of effectively processing the vast temporal extent of surgical videos is a formidable endeavor. Traditional methods commence with the development of a spatial feature extractor, which then feeds into a temporal feature extractor. However, existing strategies typically employ phase labels at the frame level for spatial feature training (Czempiel et al., 2020, Gao et al., 2020, Jin et al., 2021), an approach prone to ambiguity when distinct phases contain visually similar scenes, as illustrated in Fig. 1. This ambiguity represents a substantial obstacle to the efficient training of spatial feature extractors, highlighting the imperative for a more refined pairing of input data and supervisory signals.

Moreover, as indicated by Fig. 1, inaccurate identification of pivotal events such as ‘Clipping and Cutting’ can precipitate the misclassification of subsequent frames. This underlines the necessity for a method that reinforces the recognition of phase transitions, thereby improving the model’s capacity for understanding the entire surgical workflow.

Regarding temporal analysis, extant single-task models for surgical phase recognition fall into three broad categories: those leveraging Recurrent Neural Networks (RNNs), Convolutional Neural Networks (CNNs) (LeCun et al., 1998), and Transformers (Vaswani et al., 2017). RNNs, including Long Short-Term Memory (LSTM) networks (Hochreiter and Schmidhuber, 1997), have difficulty in capturing extensive temporal dependencies, CNN-based methods such as Temporal Convolutional Networks (TCNs) (Lea et al., 2016, Farha and Gall, 2019) apply uniform filter sizes which might not efficiently capture long-duration patterns.

To address these challenges, we propose the Long Video Transformer (LoViT), exhibiting state-of-the-art performance in handling lengthy surgical videos. Our contributions are threefold:

• We introduce a temporally-rich spatial feature extractor that transcends conventional spatial recognition paradigms by incorporating temporal awareness within the feature extraction phase, thus significantly enhancing the model’s ability to interpret complex temporal progressions in surgical procedures.
• We innovate with a phase transition-aware supervision mechanism that accentuates pivotal transitional moments within surgeries. This forward-thinking strategy equips our model with an elevated understanding of the procedural narrative inherent in surgical operations.
• Lastly, our incorporation of multi-scale temporal feature aggregation, while not the core of our contributions, still represents a critical enhancement to our model. This fusion of local and global temporal information augments the model’s robustness, ensuring that our primary advancements remain at the forefront of our work.

## 2 Related work

The evolution of surgical phase recognition models has seen a paradigm shift towards single-task learning that capitalizes exclusively on phase labels. SV-RCNet (Jin et al., 2018) integrated Residual Networks (ResNets) (He et al., 2016) for extracting spatial features and coupled them with LSTMs to encode temporal dynamics, promoting an end-to-end framework. Moreover, Gao et al. (2020) incorporated a tree search algorithm into LSTM structures to take advantage of the future context in sequence processing. Despite these advances, the limitations of LSTMs in managing extended sequences become apparent in multi-hour surgical videos (Czempiel et al., 2020). Addressing this, TMRNet (Jin et al., 2021) implemented a non-local bank operator to create connections between the current frame and the LSTM-generated features, although simplifying previous contributions to a weighted summation, which dilutes the capture of global contexts.

Changing focus, several studies (Czempiel et al., 2020, Yi et al., 2022) adopted Temporal Convolutional Networks (TCNs) as their backbone due to their ability to capture long-term dependencies. However, the use of dilated convolutions (Oord et al., 2016) in long sequences may compromise the granularity of temporal relationships, a critical challenge posed by the prolonged duration of surgical procedures.

The ascent of Transformers in NLP (Vaswani et al., 2017), particularly for their self-attention mechanisms, has revolutionized video processing tasks, such as classification (Bertasius et al., 2021, Arnab et al., 2021), language location (Zhang et al., 2021), and re-identification (Tang et al., 2022). These approaches have leveraged self-attention for frame sequences, yet they primarily cater to shorter video clips. For example, the Anticipative Video Transformer (AVT) (Girdhar and Grauman, 2021) introduced a causal layer to the ViT, aligning well with shorter sequences but not as adept for longer durations. Notably, TeSTra (Zhao and Krähenbühl, 2022) propels the field forward by enabling efficient processing of long natural video sequences for action recognition. It updates global features selectively, discarding outdated information, which is efficient but overlooks the enduring relevance of past events to update global features in the unfolding of a surgical phase.

In surgical phase recognition, the field has seen innovations like Czempiel et al.’s Transformer model (Czempiel et al., 2021), focusing on future phase anticipation. Trans-SVNet (Gao et al., 2021) aimed to tackle the limitations of TCNs noted in TeCNO (Czempiel et al., 2020) by integrating Transformers for multiscale temporal feature fusion. However, this approach introduced an extra processing stage workload and failed to fully resolve issues related to processing long-term information using dilated convolutions.

The Informer model (Zhou et al., 2021) addresses the computational intensity of Transformers on long videos with its *ProbSparse* self-attention mechanism, reducing complexity to O(Llog(L)). Our methodology extends this innovation, combining *ProbSparse* with traditional self-attention. This fusion is distinct from previous multiscale strategies (Mun et al., 2020, Fan et al., 2021), which did not resolve the complexity of processing long sequences and mainly targeted to analyze surgical videos in different views. We address this gap by applying *ProbSparse* for overarching temporal patterns and standard self-attention for local details, facilitating a comprehensive and detailed analysis of prolonged surgical procedures. Our temporal module is end-to-end trainable, promising to tackle the complexity of long-term sequence recognition without compromising the granular insights critical for surgical phase detection.

In the pursuit of advancing the domain of surgical phase recognition, we critically evaluate the paradigm that prevails in the training of spatial feature extractors in isolation. Conventional methodologies, as illustrated in the works such as Gao et al. (2021) and Jin et al. (2021), hinge upon the utilization of image-phase pairings for supervision. This traditional schema is susceptible to ambiguities, as it may not adequately address the occurrence of visually similar patterns that span disparate surgical phases. Such methods insufficiently account for surgical procedures’ inherently dynamic and temporally evolving nature, where phases unfold and transition with subtle yet critical variations. Yi and Jiang (2019) introduced the Online Hard Frame Mapper (OHFM) to address the complexity of identifying ‘hard frames’ that exhibit visual similarities but have different phase labels. While OHFM emphasizes the importance of discerning differentiation, it falls short of refining the feature extraction process itself. Instead, OHFM adds an additional time-consuming step for selecting and independently recognizing these frames, without fundamentally addressing the underlying issue. In light of these limitations, we introduce a novel temporally-rich spatial feature extractor. Our proposed methodology transcends the constraints of static image analysis by intrinsically incorporating temporal dynamics into the feature extraction phase, thus enhancing the model’s performance. This harmonized approach fosters a nuanced understanding of surgical phases, facilitating the differentiation of visually congruent but temporally disparate events, a problem not addressed in the existing literature. Our advanced temporally-rich methodology lays the foundation for a more sophisticated understanding of the continuous and evolving surgical environment. It positions our work at the forefront of the field, pioneering a more contextually enriched paradigm for surgical phase recognition that is sensitive to the chronological flow and intricate details of surgical procedures.

In addition to the methods described previously for enhancing the model’s comprehension of video inputs, our model considers the critical importance of specific events indicative of phase transitions, i.e. those key moments that delineate the progression of a surgical procedure. Inspired by the field of object detection, where Gaussian kernels are skillfully employed by algorithms such as CornerNet (Law and Deng, 2018) and CenterNet (Duan et al., 2019) to highlight salient features, we introduce an innovative phase transition map. This map uses a kernel-based approach to amplify areas denoting transitional activities, thus endowing our model with a nuanced lens for identifying crucial changes in surgical stages. This straightforward yet efficacious strategy which has been largely underestimated in prior work contributes to a more robust supervision of this task.

This two-pronged innovation, a temporal enrichment of spatial features in conjunction with a phase transition map, captures not only the overarching narrative of the surgical workflow but also the fine-grained nuances of its critical junctures. The practicality and efficacy of our proposed methodology are substantiated not solely by theoretical assertions, but by a series of stringent experimental validations. These evaluations confirm that our model achieves great enhancements in the accurate recognition of surgical phase transitions.

## 3 Methods

In this work, we target the problem of online surgical phase recognition. Formally, this is a video classification problem where we aim to solve for a mapping f such that $f_{\theta }\left(X_{t}\right)\approx p_{t}$, where $X_{t}={\left\{x_{j}\right\}}_{j=1}^{t}$ is a given input video stream, and $x_{j}\in R^{H\times W\times C}$. The symbols H, W, and C represent the image height, width, and number of channels, respectively. As in our work, we deal with RGB images, C=3. The height and width of each video frame change from dataset to dataset. The first frame of the video is noted as $x_{1}$, and the current tth frame as $x_{t}$. The output $p_{t}\in {\left\{k\right\}}_{k=1}^{K}$ is the class index corresponding to the surgical phase of the video frame $x_{t}$, where K is the total number of classes or surgical phases. The symbol θ is a vector of parameters corresponding to the weights of our network model f, which we call LoViT throughout the paper.

**Fig. 2:**
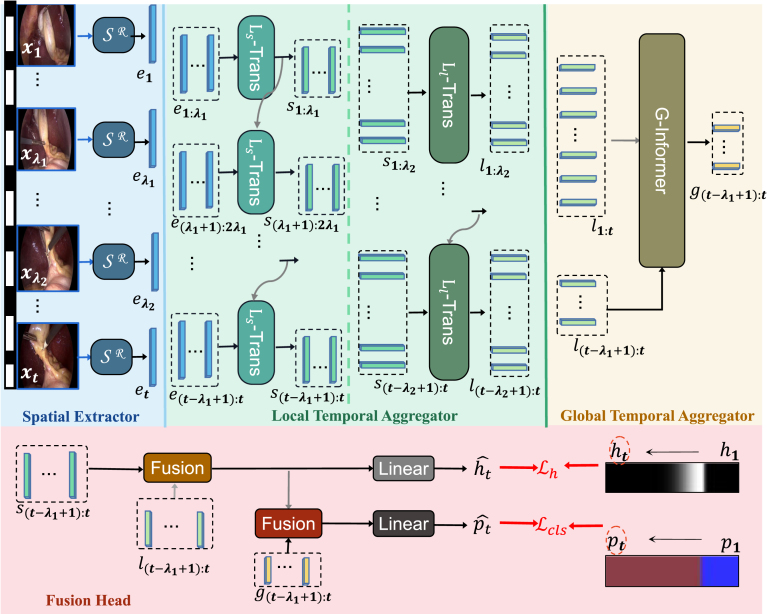
The proposed LoViT framework for surgical video phase recognition. The $S^{R}$ module extracts temporally-rich spatial features e from each video frame x. Two cascaded L-Trans modules ($L_{s}$-Trans and $L_{l}$-Trans) output local temporal features s and l with inputs of different local window sizes ($\lambda_{1}$ and $\lambda_{2}$). G-Informer captures the global relationships to generate the temporal feature g. A fusion head combines the multi-scale features s, l, and g, followed by two linear layers that learn a phase transition map ${\overset{ˆ}{h}}_{t}$ and a phase label $\overset{ˆ}{p_{t}}$ of the current tth video frame $x_{t}$. Modules with the same color share the same weight. During training, $S^{R}$ is trained separately and its weights are then frozen to train the other temporal modules of LoViT.

### 3.1 Overview of LoViT architecture

As shown in Fig. 2, our LoViT embodies a temporally-rich spatial feature extractor followed by a multiscale temporal feature aggregator. Specifically, the temporally-rich spatial feature extractor embeds surgical video frames and then feeds them to the multiscale temporal feature aggregator, which includes the Transformer-based module L-Trans for abstracting short fine-grained information, such as actions and tools, and the Informer-based module G-Informer for processing long-term information, such as key clip information of current phase and the relationship among phases. Moreover, a multiscale temporal fusion head integrates local and global features together that is used for classifying surgical phases with the support of phase transition-aware supervision.

**Fig. 3:**
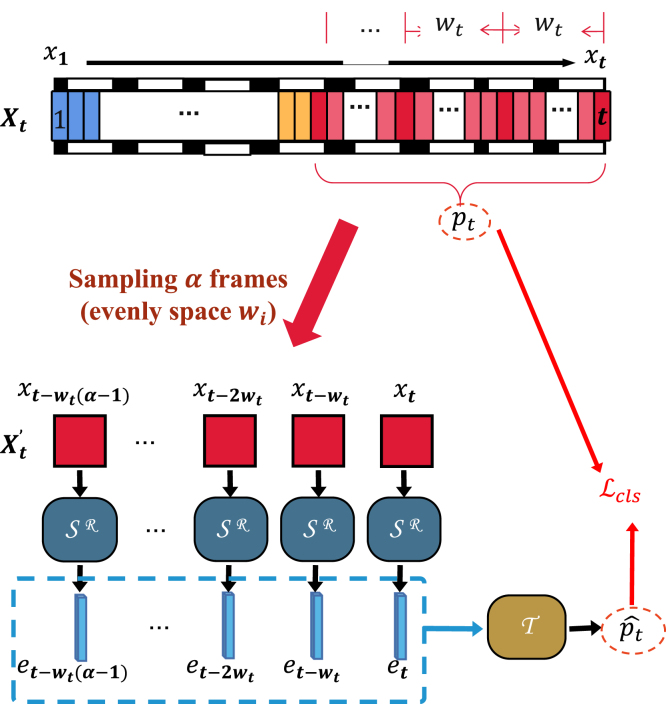
The architecture of training the temporally-rich spatial feature extractor. During the tth frame training, a video stream $X_{t}={\left\{x_{j}\right\}}_{j=1}^{t}$ is sampled at evenly spaced intervals $w_{t}$ from the start of the current phase to the current frame, producing $X_{t}^{\prime }\subseteq X_{t}$. Each frame $x\in X_{t}^{\prime }$ is embedded using the spatial feature extractor $S^{R}$, then grouped into a feature sequence (with a blue dashed box). A temporal aggregator T follows to add temporal information for recognition. The predicted phase $\overset{ˆ}{p_{t}}$ is compared to the corresponding ground truth phase $p_{t}$ to compute a cross-entropy loss. We will throw T and only retain $S^{R}$ for spatial feature extraction after the training stage.

### 3.2 Temporally-rich spatial feature extractor

Surgical videos are often several hours long, posing a formidable challenge for training models end-to-end due to the immense computational demand. To circumvent this, models are typically segmented into two distinct training steps: an initial spatial feature extraction followed by a secondary temporal feature extraction. The common practice for training the spatial feature extractor entails applying frame-level supervision. Each frame is input individually, and the model is tuned to predict the phase corresponding to that frame. This approach, while straightforward, can result in ambiguities. It is not uncommon for similar visuals to occur across various phases, causing the model to misinterpret the phase transitions (see Fig. 1). This resemblance across different phases can confuse the model, potentially leading to a situation where the spatial feature extractor is inadequately trained if solely dependent on frame-level supervision. To address these issues, we propose a novel method for training a spatial feature extractor that is enriched with temporal context. Unlike the conventional frame-to-phase mapping, we aim to establish a robust image set-to-phase relationship, as depicted in Fig. 3.

Our approach leverages a set of frames, $X_{t}^{\prime }$, selected from the sequence $X_{t}$ and processes each through the spatial feature extractor, $S^{R}$, to yield spatial features e. These features are then sequenced into $E_{t}$ and fed into a temporal aggregator, T, for phase classification. We train $S^{R}$ and T concurrently in an end-to-end fashion to produce a spatial feature extractor that captures temporal dynamics, $S^{R}$.

Given the intensive computational resources required for processing lengthy videos and the empirical observation that salient temporal information predominantly emerges at the beginning of each phase, we limit our frame selection to α=30 images. These are uniformly distributed from the start of the current phase $x_{b_{p_{t}}}$ up to the current frame $x_{t}$. To generate this subset, $X_{t}^{\prime }$, we introduce a sampling interval, $w_{t}$, determined by the equation $w_{t}=\left\lceil \frac{t-b_{p_{t}}}{\alpha }\right\rceil$. This yields a sequence of frames selected at equal intervals, ensuring that we maintain a consistent temporal resolution across the extracted phase:

(1) $$X_{t}^{\prime }=\left(x_{t-w_{t}\left(\alpha -1\right)},x_{t-w_{t}\left(\alpha -2\right)},\ldots ,x_{t}\right).$$

With this methodology, the predicted phase $\overset{ˆ}{p_{t}}$ for the tth frame becomes:

(2) $$\overset{ˆ}{p_{t}}=T\left(E_{t}\right)\;=T\left(\left(S^{R}\left(x_{t-w_{t}\left(\alpha -1\right)}\right),S^{R}\left(x_{t-w_{t}\left(\alpha -2\right)}\right),\ldots ,S^{R}\left(x_{t}\right)\right)\right).$$

Inspired by the AVT model known for its efficacy in short video action anticipation tasks, our architecture employs a ViT-based spatial feature extractor, $S^{R}$, and a causally masking attention Transformer as temporal aggregator, T. We use cross-entropy loss for model training. Post-training, T is discarded, leaving $S^{R}$ with its fixed weights ready to convert each frame, x, into a spatial feature vector, e. These vectors serve as input to the subsequent temporal feature aggregator.

This tailored approach to frame selection, which we rigorously tested through various experiments, proved not only to be efficient in training due to reduced memory requirements but also effective in maintaining high model performance, comparable to that achieved using longer frame sequences.

### 3.3 Local temporal feature aggregator

**Fig. 4:**
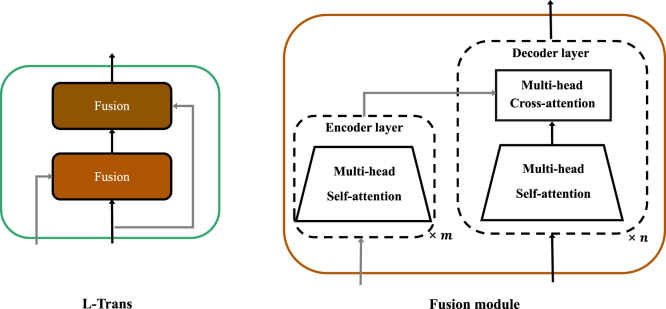
**L-Trans:** The L-Trans adopts two cascaded fusion modules to process two-branch temporal inputs (grey line and black line). **Fusion module:** It consists of an encoder and a decoder. The encoder is comprised of an m-layer self-attention layer for the grey line input, and the decoder is composed of an n-layer cascaded self-attention with cross-attention for processing the encoder’s output and the black line input.

We designed a local Transformer-based temporal feature aggregator (**L-Trans**) to extract local fine-grained temporal information, which is visualized in Fig. 4. L-Trans begins by analyzing the current local temporal features with the assistance of the previous clip’s output, using a fusion module. A second fusion module then refines the output by incorporating initial current features. Fig. 4 also depicts the fusion module, which consists of an m-layer self-attention encoder for auxiliary features and an n-layer cascaded self-attention module with cross-attention for the encoder’s output and the decoder branch input.

The self-attention mechanism reduces the length between every network signal to the shortest O(1) through the dot-product computation between every two signals and avoids the recurrent structure, whereby the Transformer is used for temporal feature aggregation. The self-attention mechanism (Vaswani et al., 2017), is formulated as:

(3) $$A\left(Q,K,V\right)=\mathrm{Softmax}\left(\frac{QK^{T}}{\sqrt{d_{k}}}\right)V,$$

where $Q\in R^{L_{Q}\times d}$, $K\in R^{L_{K}\times d}$, $V\in R^{L_{V}\times d}$, and d is the input dimension. Q, K, and V represent the standard matrices referred to as query, key, and value in Transformer-based architectures, respectively. In contrast, cross-attention refers to the attention mechanism that takes into account both the query and key input from different sources.

To capture the nuances of local temporal dynamics at various scales, we utilize a pair of cascading L-Trans modules: the smaller-scale $L_{s}$-Trans and the larger-scale $L_{l}$-Trans, each ingesting sequences of lengths $\lambda_{1}$ and $\lambda_{2}$ respectively. The process begins with the spatial feature ensemble e, derived from our sophisticated temporally-rich spatial feature extractor. This set flows through $L_{s}$-Trans to generate the refined small-scale local feature set s. We incorporate feedback by feeding the preceding output of $L_{s}$-Trans back into the system. Analogously, the sequence s is then input into $L_{l}$-Trans to obtain the broad-scale local feature set l, with the process also utilizing feedback from its previous output.

Specifically, our method employs a sliding window technique for local feature processing. For training efficiency and memory optimization, we compute gradients solely for the last clip in the sequence when recognizing the tth frame, thereby disregarding preceding gradients. This selective gradient calculation has been rigorously tested and proven to deliver equivalent performance outcomes while facilitating a more efficient training regimen. A salient distinction is drawn between the training and inference stages; the latter necessitates reprocessing all antecedent frames to discern the current frame, but permits the reuse of previously computed features, thereby conserving computational exertions.

### 3.4 Global temporal feature aggregator

Despite the self-attention mechanism inherent in Transformer-based architectures has the capability of extracting temporal relationships (see Eq. (3)), it requires quadratic dot-product $O\left(L_{Q}L_{K}\right)$ time computation and memory usage, which limits the processing of long sequences. To overcome this limitation, we propose the use of Informer (Zhou et al., 2021), which consists of a more efficient implementation of the self-attention mechanism called *ProbSparse*, whereby only a few dot-product pairs contribute to the major attention. *ProbSparse* reduces time and memory usage to O(Lln(L)) where L represents the length of the input sequence. *ProbSparse* is formulated as:

(4) $$A_{PS}\left(Q,K,V\right)=\mathrm{Softmax}\left(\frac{\bar{Q}K^{T}}{\sqrt{d_{k}}}\right)V,$$

where $q_{i}$ represents the ith row in Q, and $\bar{Q}$ is a sparse matrix of the same size as q, containing only the top u queries under the sparsity max-mean measurement:

(5) $$\bar{M}\left(q_{i},K\right)=\underset{j}{\max}\left(\frac{q_{i}k_{j}^{T}}{\sqrt{d_{k}}}\right)-\frac{1}{L_{K}}\overset{L_{K}}{\underset{j=1}{\sum }}\frac{q_{i}k_{j}^{T}}{\sqrt{d_{k}}}.$$

Under the long-tail distribution, we randomly sample $U=L_{K}\ln\left(L_{Q}\right)$ dot product pairs to calculate $\bar{M}\left(q_{i},K\right)$, and fill the rest with zeros.

We designed a Global Temporal Informer (**G-Informer**) to capture long-range dependencies more efficiently. The G-Informer framework contains two branches for processing two types of inputs: a long local feature sequence $\left(l_{1},\ldots ,l_{t}\right)$ and the current local feature $\left(l_{t-\lambda_{1}+1},\ldots ,l_{t}\right)$. Unlike the fusion model used for L-Trans, the first input branch of G-Informer for the long sequence uses the *ProbSparse* self-attention mechanism.

### 3.5 Multiscale temporal feature fusion head.

Feature down-sampling and sparse attention, unfortunately, lead to the loss of fine-grained characteristics in G-Informer. To overcome this limitation, we employ a multiscale temporal feature fusion head to combine the local (small and large) and global features from L-Trans and G-Informer, respectively. As illustrated at the bottom of Fig. 2, the multiscale head contains two fusion modules. The first is to merge the short and long local temporal features, $\left(s_{t-\lambda_{1}+1},\ldots ,s_{t}\right)$ and $\left(l_{t-\lambda_{1}+1},\ldots ,l_{t}\right)$, obtained from $L_{s}$-Trans and $L_{l}$-Trans, respectively. Subsequently, another fusion module is utilized to merge the fused local features with the global temporal features $\left(g_{t-\lambda_{1}+1},\ldots ,g_{t}\right)$ obtained from G-Informer.

### 3.6 Phase transition-aware supervision.

**Fig. 5:**
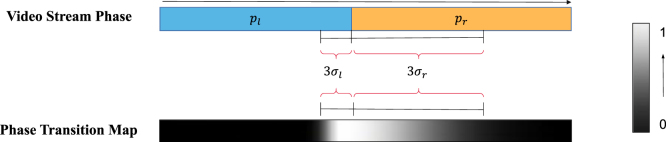
The example of building phase transition map. We project phase transition area onto a phase transition map using a left–right asymmetric Gaussian kernel where left- and right-side kernel lengths are $3\sigma_{l}$ and $3\sigma_{r}$ respectively. $p_{l}$ and $p_{r}$ mean adjacent different phases.

To be able to enforce that our model knows how the video shifts from one phase to another phase, which requires a stronger understanding of the surgery workflows, we cast transition frames – those situated next to standard phase frames – onto a phase transition map denoted by h. This mapping employs a one-dimensional asymmetric Gaussian kernel, the visualization of which is depicted in Fig. 5. This selective emphasis on transition frames is crucial for our proposed Learning over Video Transitions (LoViT) model to detect the nuanced changes that characterize different surgical phases.

The core principle behind the phase transition map h is to encapsulate the area of a phase transition within a Gaussian distribution. This enables the model to focus intensively on the transition zones, with a gradual reduction in attention as the frames move away from the transition point. The value assigned to the tth frame on this map is expressed as follows:

(6) $$h_{t}=\left\{\begin{array}{cc} \exp\left(-\frac{{\left(t-b_{p_{t}}\right)}^{2}}{2\sigma_{l}^{2}}\right),\; & b_{p_{t}}-3\sigma_{l}<t<b_{p_{t}}\\ \exp\left(-\frac{{\left(t-b_{p_{t}}\right)}^{2}}{2\sigma_{r}^{2}}\right),\; & b_{p_{t}}<t<b_{p_{t}}+3\sigma_{r}\\ 0, & \;otherwise \end{array}\right)$$

The asymmetric nature of the Gaussian kernel, with lengths $3\sigma_{l}$ and $3\sigma_{r}$ on the left and right sides respectively, originates from $b_{p_{t}}$—the frame where the phase transition is pinpointed. In this scheme, the Gaussian kernel is centered at the transition point, where its value peaks at 1, signifying the highest importance. As one moves away from the center, the importance diminishes, following the Gaussian curve, until it reaches zero outside the designated transition area. This results in a focused region where the model’s attention is heightened, encapsulating the transition between phases with high precision.

Incorporating this intricate mapping into our LoViT model’s learning process is accomplished through a custom loss function. This function is a composite of two distinct losses: the phase-transition map loss and the phase class loss. Mathematically, it is represented as:

(7) $$L^{*}=L_{1}\left(\overset{ˆ}{h},h\right)+L_{CE}\left(\overset{ˆ}{p},p\right),$$

where the first term $L_{1}\left(\overset{ˆ}{h},h\right)$ quantifies the $L_{1}$ loss comparing the predicted phase transition map $\overset{ˆ}{h}$ against the actual ground truth h. This term ensures that the model’s sensitivity to the transition areas is fine-tuned. The second term, $L_{CE}\left(\overset{ˆ}{p},p\right)$, is the cross-entropy loss which assesses the model’s proficiency in accurately predicting the surgical phase given the ground truth p. This dual-loss configuration is instrumental in harmonizing the transition detection with the accurate classification of phases, thereby elevating the model’s overall performance in handling surgical video data.

The proposed framework underscores our commitment to advancing a nuanced, context-aware model capable of interpreting the complex temporal dynamics of surgical procedures.

## 4 Experimental design

### 4.1 Datasets.

We extensively performed experiments on two publicly available surgical video datasets, namely Cholec80 (Twinanda et al., 2017) and AutoLaparo (Wang et al., 2022), capturing cholecystectomy and hysterectomy surgical interventions, respectively. Cholec80 (Twinanda et al., 2017) consists of 80 high-resolution, of either 1920 × 1080 or 854 × 480 pixels, laparoscopic surgical videos with an average video duration of 39 min at 25 frames-per-second (fps). These databases are provided with manual annotations done by surgeons indicating the surgical phase each video frame belongs to and the tools appearing in the scene. Cholec80 videos consist of seven phases. For this study, we only used phase annotations. For a fair comparison with previous methods (Jin et al., 2018, Twinanda et al., 2017, Yi and Jiang, 2019, Gao et al., 2021), we keep intact the splitting of the dataset into first 40 videos for training and the remaining 40 videos for testing. AutoLaparo (Wang et al., 2022) consists of 21 videos with 7 phases, recorded at 25 Hz of a resolution of 1920 × 1080 pixels with an average video duration of 66 min. We split the dataset into 10 videos for training, 4 videos for validation, and 7 videos for testing following Wang et al. (2022). Similar to other works presented in the literature (Jin et al., 2018, Twinanda et al., 2017, Gao et al., 2021), we sampled both datasets into 1 fps and resized the frame size to 250 × 250 pixels.

### 4.2 Implementation details

Our method is implemented with the PyTorch framework (Paszke et al., 2019). All experiments were carried out on an Intel Xeon W-2195 CPU (2.3 GHz), 125 GB RAM, and a single NVIDIA Tesla V100 GPU. Following the AVT model (Girdhar and Grauman, 2021), our temporally-rich spatial feature extractor is a 12-head, 12-layer Transformer encoder model that uses the ViT-B/16 architecture, which is pre-trained on ImageNet-1K (IN1k) (Deng et al., 2009) with input image size of 248 × 248 pixels and output size of $D_{s}=768$D representations. We trained the feature extractor with SGD+momentum for 35 epochs, with a 5-epoch warmup (Goyal et al., 2017) and cosine annealed decay. The rest of LoViT was trained for 50 epochs with SGD+momentum, weight decay of 1e−5, learning rate of 3e−4, and a 5-epoch warm-up and 45-epoch cosine annealed decay. During experiments, LoViT was fed 3000 video frames and produced outputs with dimensions of 512, 64, and 8 for $L_{s}$-Trans, $L_{l}$-Trans, and G-Informer, respectively. Both $L_{s}$-Trans and $L_{l}$-Trans have a fusion module, each with a 2-layer encoder and a 2-layer decoder. G-Informer has a 2-layer encoder and a 1-layer decoder. The multiscale head fusion modules have a two-layer encoder and a one-layer decoder. To balance computational demands and the accuracy of temporal aggregation, we computed gradients for $L_{s}$-Trans using the last $\lambda_{1}=100$ frames and the gradients for $L_{l}$-Trans using the last $\lambda_{2}=500$ frames. About the proposed phase transition map, we set $\sigma_{l}=3$ and $\sigma_{r}=12$.

### 4.3 Evaluation

In this paper, we investigate the performance of LoViT in comparison to state-of-the-art approaches, followed by extensive ablation experiments to demonstrate the effect its different components have on a surgical phase recognition task. According to previous work, we use four commonly used measures in surgical phase recognition, namely accuracy (AC), precision (PR), recall (RE), and Jaccard (JA). AC refers to the percentage of correctly recognized frames and is video-based. However, the video class is imbalanced, and the short phases have little impact on the whole video’s accuracy. Therefore, we further report class- (phase-) level precision, recall, and Jaccard, which represent positive predictive value, positive rate, and intersection rate of recognition versus ground truth, respectively.

Previous work (Gao et al., 2021, Jin et al., 2021, Yi et al., 2022) reported the relaxed metric^1^ in their papers, which was originally introduced in the MICCAI 2016 M2CAI Challenge^2^. This metric considers incorrect predictions those that are not equal to the ground truth but fall into neighboring phases as correct within a 10-second window around the phase transition. However, phase transition prediction is also an important indicator of a model’s ability. We included this approach in experiments comparing with state-of-the-art methods to ensure a fair comparison with previous techniques. Furthermore, we consistently utilized the standard metric in all ablation studies.

## 5 Results

### 5.1 Comparison with state-of-the-art methods

To assess the effectiveness of our proposed method, we carried out a comparative study, contrasting our LoViT model against contemporary state-of-the-art techniques pertinent to the domains of action anticipation and surgical phase recognition. This study utilized two distinct datasets: Cholec80 (Twinanda et al., 2017) and AutoLaparo (Wang et al., 2022).

**Table 1:** The results (%) of different state-of-the-art methods on both the Cholec80 and AutoLaparo datasets. The best results are marked in bold.

Dataset	Method	Stage	Relaxed metric	Video-level Metric	Phase-level Metric			Parameters
				Accuracy↑	Precision↑	Recall↑	Jaccard↑	
Cholec80	PhaseNet (Twinanda et al., 2016)	2	✓	78.8±4.7	71.3±15.6	76.6±16.6	–	58.3 M
	SV-RCNet (Jin et al., 2018)	2	✓	85.3±7.3	80.7±7.0	83.5±7.5	–	28.8 M
	OHFM (Yi and Jiang, 2019)	3	✓	87.3±5.7	–	–	67.0±13.3	47.1 M
	TeCNO (Czempiel et al., 2020)	2	✓	88.6±7.8	86.5±7.0	87.6±6.7	75.1±6.9	24.7 M
	TMRNet (Jin et al., 2021)	3	✓	90.1±7.6	90.3±3.3	89.5±5.0	79.1±5.7	–
	Trans-SVNet (Gao et al., 2021)	3	✓	90.3±7.1	90.7 ± 5.0	88.8±7.4	79.3±6.6	24.7 M
	Not End-to-End (Yi et al., 2022)	3	✓	91.5±7.1	–	86.8±8.5	77.2±11.2	–
	LoViT (ours)	2	✓	92.4 ± 6.3	89.9±6.1	90.6 ± 4.4	81.2 ± 9.1	26.8 M
	Trans-SVNet	3		89.1±7.0	84.7 ± 7.6	83.6±6.6	72.5±7.5	24.7 M
	AVT (Girdhar and Grauman, 2021)	1		86.7±7.6	77.3±9.8	82.1±5.5	66.4±10.0	307.4 M
	TeSTra (Zhao and Krähenbühl, 2022)	2		90.4±6.6	82.8±8.5	83.8±7.4	71.6±10.1	27.6 M
	LoViT (ours)	2		91.5 ± 6.1	83.1±9.3	86.5 ± 5.5	74.2 ± 11.3	26.8 M

AutoLaparo	SV-RCNet	2		75.6	64.0	59.7	47.2	28.8 M
	TMRNet	3		78.2	66.0	61.5	49.6	–
	TeCNO	2		77.3	66.9	64.6	50.7	24.7 M
	Trans-SVNet	3		78.3	64.2	62.1	50.7	24.7 M
	AVT	1		77.8	68.0	62.2	50.7	307.4 M
	LoViT (ours)	2		81.4 ± 7.6	85.1 ± 9.9	65.9 ± 30.7	56.0 ± 25.7	26.8M

In the upper part of Table 1, we present a quantitative comparison of the Cholec80 dataset. It should be noted that we re-implemented Trans-SVNet using the weights made available by the authors of the original study. As for AVT, our implementation was based on the last 30 frames according to the code released along with their publication. The reported results of other benchmark methods were directly cited from their respective publications. Methods such as OperA (Czempiel et al., 2021) were not included in our comparison due to discrepancies in dataset splits and the absence of publicly accessible code. Additionally, we excluded SAHC (Ding and Li, 2022) on account of an evaluative oversight: they utilized a frame rate of 25fps instead of the 5fps used for their ground truth^3^. Table 1 reveals that our LoViT model surpasses other methods in the majority of the evaluated metrics, with the sole exception being precision on the Cholec80 dataset. Specifically, LoViT attained an accuracy that exceeds the benchmark set by Trans-SVNet by a margin of 2.4 pp. Moreover, our model demonstrates superior performance over AVT, the leading model for action anticipation, by a difference of 4.8 pp in accuracy. It also exhibits more consistent performance, as evidenced by a reduced standard deviation in accuracy by roughly 1 pp in contrast to Trans-SVNet. Furthermore, LoViT showcased better results when comparing our temporal module to a re-implementation of the temporal module proposed for long-term action recognition in TeSTra (Zhao and Krähenbühl, 2022). Beyond standard metrics, LoViT also proved to be more effective when evaluated against relaxed metrics 1.

The quantitative comparison on the AutoLaparo dataset is depicted in the bottom part of Table 1. The results suggest that surgical phase recognition is more challenging on AutoLaparo, compared to Cholec80, since it is representative of a more complex workflow constrained by a smaller dataset size. We Ref. Wang et al. (2022) to populate Table 1 with existing methods evaluated on this dataset. LoViT consistently outperformed in both video-level and phase-level metrics. We observe that TMRNet (Jin et al., 2021), TeCNO (Czempiel et al., 2020), and Trans-SVNet (Gao et al., 2021) perform similarly on this dataset with average accuracy of 77%. Compared to the state-of-the-art method Trans-SVNet, we observed LoViT had a higher performance with a 3.1 pp accuracy margin. Besides video-level accuracy, we highlight that reported phase-level metrics are crucial due to the imbalance of phase distribution. Compared with Trans-SVNet, an increase of 20.9 pp (64.2% → 85.1%), 3.8 pp (62.1% → 65.9%), and 5.2 pp (50.7% → 55.9%) margins were achieved using LoViT in relation to precision, recall, and Jaccard, respectively.

**Fig. 6:**
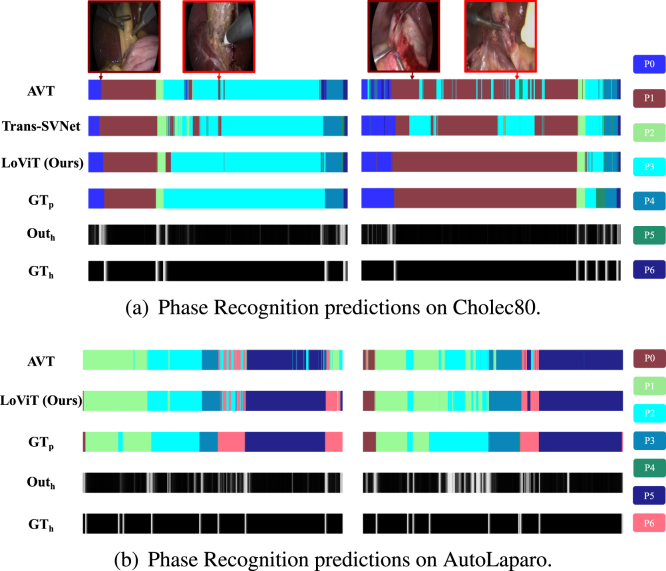
Qualitative comparisons with some other methods in the Cholec80 and AutoLaparo datasets. The first line in (a) presents some images in the video corresponding to the moment pointed by the red arrow, where light red presents incorrect examples of both AVT and Trans-SVNet, and dark red presents wrong examples of only Trans-SVNet. The following four lines in (a) and the first three lines in (b) represent the phase results recognized by different methods and the corresponding ground truth ${\mathrm{GT}}_{p}$. The last two lines in both subfigures mean the heatmap output from the proposed LoViT $\overset{ˆ}{h}$ and its Ground Truth ${\mathrm{GT}}_{h}$.

To illustrate the performance of our approach compared to the state of the art, in Fig. 6 we present a qualitative comparison of two examples drawn from the Cholec80 and AutoLaparo test datasets. As observed in Fig. 6(a), for some ambiguous frames shown (first row), Trans-SVNet was unable to effectively classify the correct phase. Although the surgical phases of laparoscopic cholecystectomy are executed linearly, video frames are misclassified into strictly nonlinear phases. On the contrary, LoViT learned a better long-term temporal context than Trans-SVNet, even when a few misclassifications are still nonlinear. For some situations (first row with dark red box), Trans-SVNet even performs worse than AVT, which inputs short video clips. This further proves that Trans-SVNet loses some fine-grained and continuity information while processing long videos.

When investigating the performance in learning the heatmap capturing phase transitions, we observed that LoViT’s performance is highly accurate compared to the ground truth, as shown in the last two rows of Fig. 6(a,b). In our ablation studies, we demonstrate the benefit of including heatmap information, since it further helps to extract relationships among phases.

### 5.2 Inference time analysis

**Fig. 7:**
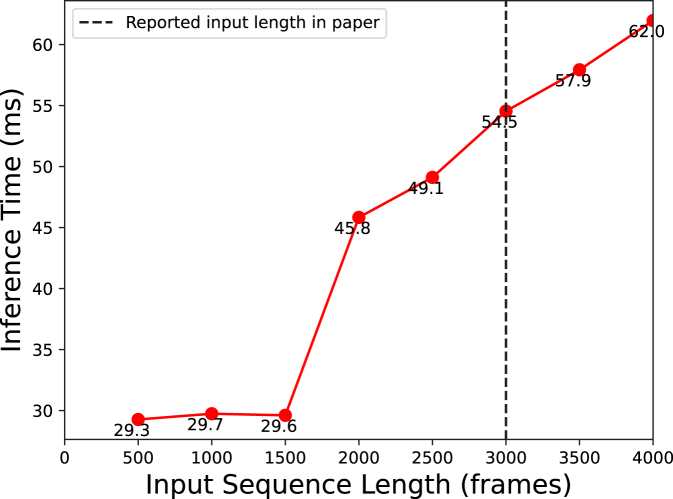
Inference time visualization of LoViT for different input video lengths.

To demonstrate the efficacy of our proposed LoViT model’s near-linear inference time when handling extensive input sequences, we conducted experiments with videos of varying lengths and recorded the inference times, as depicted in Fig. 7. The results demonstrate that for sequences shorter than approximately 1500 frames, the inference time is largely governed by the local temporal module, efficiently processing shorter sequences. Beyond this threshold of 1500 frames, the inference time becomes predominantly influenced by the G-Informer component of our model, which efficiently leverages *ProbSparse* attention for extended sequence processing. The data reveals that the growth in inference time does not exhibit quadratic complexity but remains near-linear, substantiating our model’s capability to efficiently process prolonged video inputs. It is important to note that our objective was not to outpace the inference speed of prior state-of-the-art methods, such as Trans-SVNet, which reports an inference time of 10 ms per frame. Instead, our goal was to validate the practicality of employing an attention mechanism to effectively analyze long-duration surgical videos.

**Table 2:** The results (%) of different parts of proposed LoViT on both the Cholec80 and the AutoLaparo datasets. The best results are marked in bold.

Dataset	Model	Video-level Metric	Phase-level Metric		
		Accuracy↑	Precision↑	Recall↑	Jaccard↑
Cholec80	L-Trans	90.8±5.9	82.5	86.5	72.9
	G-Informer	91.5 ± 5.8	83.3	87.0	74.6
	LoViT	91.5 ± 6.1	83.1	86.5	74.2

AutoLaparo	L-Trans	80.6±6.9	69.7	65.1	54.0
	G-Informer	79.8±7.3	70.3	63.6	53.4
	LoViT	81.4 ± 7.4	85.1	65.9	55.9

### 5.3 Evaluating lovit architecture performance

We conducted experiments to measure the contributions of the three modules that comprise our proposed model, LoViT. These modules are: (1) Local Temporal Feature Aggregator (L-Trans), (2) Global Temporal Feature Aggregator (G-Informer), and (3) Multi-scale Temporal Feature Fusion Module (MF-Trans). Specifically, we conducted the following experiments:

• **L-Trans**: We evaluated the performance of the time aggregation model when it only contained the L-Trans module to capture local fine-grained features without the integration of global relationships.
• **G-Informer**: We evaluated the performance of a model that included time aggregation by the G-Informer module after the L-Trans. The coarse-grained information resulting from G-Informer was then directly fed into the classifier.
• **LoViT**: We evaluated the performance of our full LoViT model, which includes both the temporal local and global Transformers, followed by a multiscale temporal fusion head.

The results of the quantitative experiment are shown in Table 2. Based on the results of L-Trans and G-Informer, it can be observed that G-Informer performed better on the Cholec80 dataset, while L-Trans performed better on the AutoLaparo dataset. This suggests that global relationships are more helpful for recognizing videos in Cholec80, while fine-grained features are more critical for AutoLaparo. This finding is consistent with the fact that most videos in AutoLaparo contain recurring phases, while the workflow in Cholec80 videos is much more linear. Thus, global relationships are more critical for Cholec80, while fine-grained features are more critical for AutoLaparo, as indicated by the experimental results. Comparing the performance of LoViT with that of L-Trans and G-Informer, we can see that LoViT performs similarly with G-Informer on Cholec80, but outperforms the other two models when evaluated on AutoLaparo. This suggests that while global relationships are important for Cholec80, fine-grained features are crucial for AutoLaparo. Furthermore, the feature map g produced by G-Informer is more prone to loss of fine-grained information than l, which is extracted by L-Trans. However, LoViT overcomes this limitation through the multi-scale fusion of l and g, allowing the analysis of every frame from different dimensions and resulting in improved recognition accuracy and greater stability.

### 5.4 Evaluating temporally-rich spatial feature extractor performance

**Fig. 8:**
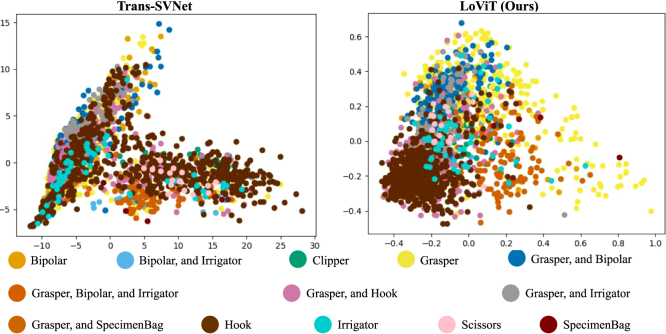
Visualization for the spatial feature distribution of different extractors. Point set: Video frames of Video 60 in Cholec80. Different colors: different tool annotations. First column: the spatial feature distribution of the frame-only spatial feature extractor in Trans-SVNet. Second column: the spatial feature distribution of the temporally-rich spatial feature extractor in our LoViT.

**Fig. 9:**
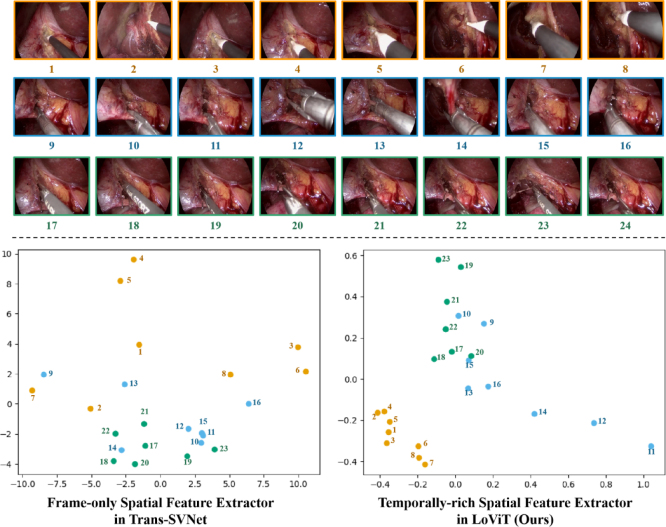
Examples of spatial feature distribution of similar video frames. *Top:* three rows depict each frame that is similar, i.e. in regards to the tool environment. *Bottom:* Visualization of the spatial feature distribution of example images using two different extractors.

We investigated the impact of our temporally-rich spatial feature extractor. The results in Table 3 demonstrate that using video clips as inputs for training temporally-rich spatial feature extractor contributes to higher performance, where the extractor inputs every single frame separately. This increase in performance is particularly notable on the AutoLaparo dataset, where we observe an improvement margin of 1.9 pp in accuracy. It is worth noting that even after replacing the temporally-rich spatial feature extractor with a regular spatial feature extractor, LoViT still surpasses current state-of-the-art methods, such as Trans-SVNet, by 1.6 pp and 1.2 pp on Cholec80 dataset and AutoLaparo dataset respectively. To better understand the superiority of our temporally-rich spatial feature extractor compared to Trans-SVNet, we conducted a principal component analysis (PCA) (Hotelling, 1933) on their feature space and projected them into a two-dimensional reduced space representation for visualization. In Fig. 8, we plotted the 2-dim feature representation of all frames in a video, labeled by the instruments appearing in each frame. We observed that our proposed temporally-rich spatial feature extractor was more effective in differentiating between different tools than the frame-only training method used in Trans-SVNet, as there was greater distinction between labeled frames in the reduced space. However, using tool labels to describe spatial features is insufficient for studying feature representations since other objects appearing in a scene, such as organs, may also affect image features. To further investigate this, we manually selected three groups of illustrative frames with similar organ contexts and grouped them by the instrument appearing in the scene. We then visualized their spatial feature distributions in Fig. 9, providing further evidence of the superiority of our method over previous spatial feature extractors.

**Table 3:** Effects (%) of Temporally-rich spacial feature extractor (R) on Cholec80 and AutoLaparo datasets. The best results are marked in bold.

Dataset	R	Video-level Metric	Phase-level Metric		
		Accuracy↑	Precision↑	Recall↑	Jaccard↑
Cholec80		90.7±6.9	80.8	85.2	71.4
	✓	91.5 ± 6.1	83.1	86.5	74.2

AutoLaparo		79.5±8.4	79.61	64.9	53.7
	✓	81.4 ± 7.4	**85.1**	**65.9**	**55.9**

### 5.5 Evaluating phase transition-aware supervision performance

We evaluate the influence of our proposed heatmap for better learning phase transitions of surgical videos. Table 4 illustrates the improvement in our model’s performance on both datasets when we used a supervised phase transition map to capture the phase transition areas. These findings emphasize the importance of phase transition areas in surgical videos, as they contain critical information for identifying the start and end of each phase. By capturing these transitions, we gain a better understanding of phase relationships, which ultimately helps in reducing confusion between similar clips.

**Table 4:** Effects of adding phase transition-aware supervision on video- and phase-level metrics (%) when evaluated on Cholec80 and AutoLaparo datasets. Note that ‘✓’ means adding phase transition-aware supervision.

Dataset	Phase Transition-aware	Video-level Metric	Phase-level Metric		
		Accuracy↑	Precision↑	Recall↑	Jaccard↑
Cholec80		90.1±6.0	82.2	84.6	71.7
	✓	91.5 ± 6.1	83.1	86.5	74.2

AutoLaparo		77.9±7.9	71.0	64.8	52.6
	✓	81.4 ± 7.4	85.1	65.9	55.9

### 5.6 Discussion

In our investigation, we have found that the conventional training of spatial feature extractors for surgical phase recognition typically relies solely on image-level supervision. However, the evidence from our research points to the importance of incorporating temporal information into the spatial feature extractor to enhance the accuracy of the recognition network. We evaluated the performance of our temporally-rich spatial feature extractor against the traditional image-only supervision approach using two distinct datasets. The results conclusively showed that our model, which utilizes video clips as input, substantially outperforms the conventional image-based training method. This finding is particularly relevant in the context of surgical videos, where many frames may exhibit similar characteristics across various phases due to the subtle nature of scene changes and the often limited visibility of surgical tools. Fig. 1 visually represents this challenge by displaying scenes with similar spatial characteristics that appear during different phases (classes). This similarity can cause confusion to a standard extractor and potentially lead to overfitting, since the same spatial features are assigned different labels during the training process. Acknowledging that informative features frequently arise at the beginning of each phase, highlighting key moments of operational change, we devised a method to select a fixed number of frame images from the start of the current phase to the current frame under consideration for classification. These are spaced at regular intervals with the objective of retaining crucial temporal information. The key insight is that by adopting this strategy, our model leverages the inherent temporal cues necessary to discern the phase associated with the current frame, while operating within the bounds of available memory capacity. This selective frame sampling method allows our temporally-rich spatial feature extractor to effectively capture the essence of temporal progression, which is vital for accurate phase recognition. It ensures that, despite memory limitations, our model can still maintain a high level of performance by focusing on the most pertinent temporal information necessary for reliable phase classification.

We recognize that actions with similar appearances may appear in multiple surgical phases. The length and complexity of surgical procedures require a robust model capable of parsing extensive sequences to distil critical temporal relationships. Despite the prowess of leading-edge methods such as TeCNO (Czempiel et al., 2020) and Trans-SVNet (Gao et al., 2021) in the management of long-duration videos via Temporal Convolutional Networks (TCNs) (Lea et al., 2016), the intrinsic nature of their dilated temporal aggregation fails in maintaining a granular temporal analysis. The dilated convolutions in these methods utilize a straightforward subsampling strategy to reduce the data volume, which, albeit helpful, is indiscriminate, potentially neglecting vital temporal details. In our Transformer-based approach, we refined temporal feature aggregation by integrating vanilla self-attention for concise video segments alongside *ProbSparse* self-attention for comprehensive long-range analysis. Our method consistently outperforms TeCNO and Trans-SVNet, evidenced by our empirical success in scenarios where Trans-SVNet lags behind AVT, as depicted in Fig. 6. This comparative advantage is attributable to our model’s capacity to preserve the continuity and granularity of temporal features, offering a more accurate surgical phase recognition. Therefore, our findings advocate that the vanilla and *ProbSparse* self-attention mechanisms employed in our study have superior efficacy over TCNs in this domain, confirming their ability for detailed and cohesive temporal understanding in surgical video analysis.

Surgical videos are characterized by time dependency among phases, and pinpointing the phase transition areas is essential for discovering such dependency. To the best of our knowledge, we are the first to represent phase transitions with a phase-transition map, which is utilized to supervise the model. From the experimental results, we observe that this phase transition map improves the performance of our proposed model. We notice that phase transition-aware supervision is an easy operation and does not impose an additional burden on the model.

Taking into account the experimental results on Cholec80 and AutoLaparo datasets, we observe that AutoLaparo is more challenging than Cholec80. However, we demonstrate that LoViT considerably outperformed all state-of-the-art models. Specifically, we proved that local information is more valuable than global relationship in the experimental results on AutoLaparo, since videos are characterized by more complex phase relationships resulting from repeated phases, whereas phase relationships in Cholec80 are mostly linear with no repetition. This limitation is exacerbated by the small sample size of AutoLaparo, which results in models having lower performance when compared to their performance observed in Cholec80.

However, our proposed method has several limitations. Although LoViT shows superior performance, it remains difficult for LoViT to accurately recognize some phases that appear in an unusual operation process. As shown in Fig. 6(a), the second video exhibits poorer performance compared to the left video. This discrepancy is primarily manifested in the prediction error of phase ‘P5’. In most videos, ‘P5’ typically follows ‘P4’, but in this particular video, it precedes ‘P4’, posing difficulty for accurate recognition by LoViT and other recognition methods. We believe future research on online surgical phase recognition will focus more on discovering complex relationships between phases, an area we envisage continuing to investigate. Furthermore, despite the efficiency of LoViT’s *ProbSparse* self-attention mechanism, it still requires feeding all previous spatial features into the temporal model for recognition of each current frame. As the duration of the surgery increases, the inference speed of LoViT will deteriorate if it takes all video frame input. An ideal method, however, should avoid redundant calculations by utilizing the previous analysis results, thereby reducing time and memory costs, improving the stability of the system’s inference speed, and enabling the processing of videos of any length, which aligns with our research direction.

## 6 Conclusions

We propose a new surgical phase recognition method named LoViT, which adopts video-clip level supervision to train a temporally-rich spatial feature extractor first and then combines local fine-grained and global information via a multiscale temporal feature aggregator supported by phase transition maps. Compared to previous methods, our Transformer-based LoViT allows for efficient and robust phase recognition of long videos without losing local or global information. Moreover, our LoViT is the first to demonstrate that phase-transition maps are useful for identifying the relationships between phases. The proposed LoViT achieves state-of-the-art performance with improvements over existing methods.

## CRediT authorship contribution statement

**Yang Liu:** Conceptualization, Data curation, Formal analysis, Funding acquisition, Investigation, Methodology, Validation, Visualization, Writing – original draft. **Maxence Boels:** Methodology, Writing – original draft, Writing – review & editing. **Luis C. Garcia-Peraza-Herrera:** Methodology, Writing – original draft. **Tom Vercauteren:** Methodology, Writing – review & editing. **Prokar Dasgupta:** Methodology, Supervision, Writing – review & editing. **Alejandro Granados:** Conceptualization, Methodology, Project administration, Supervision, Validation, Visualization, Writing – original draft, Writing – review & editing. **Sébastien Ourselin:** Formal analysis, Funding acquisition, Investigation, Methodology, Project administration, Resources, Supervision, Validation, Visualization, Writing – original draft, Writing – review & editing.

## Declaration of competing interest

The authors declare the following financial interests/personal relationships which may be considered as potential competing interests: Yang Liu has patent Video Labelling of Surgical Video pending to UK Intellectual Property Office.

## Data Availability

Data will be made available on request.

## Footnotes

1 Refer to the code: https://github.com/YuemingJin/TMRNet/blob/main/code/eval/result/matlab-eval/Evaluate.m.

2 http://camma.u-strasbg.fr/m2cai2016/.

3 As indicated on https://github.com/xmed-lab/SAHC, gt = gt[::5]’ in prototype.py’ generates a 5fps output, in contrast to fps = 25’ in Main.m’. This discrepancy, which we confirmed with the authors, inadvertently widens the relaxed boundary by a factor of five and inflates the metric value by approximately 1 percentage point (pp).
